# Bibliographical

**Published:** 1890-07

**Authors:** 


### Bibliographical.

"A critical examination of the teeth of several races, including one hundred and fifty Mound Builders, selected from the collection of the Army Medical Museum, at Washington, D. C., by E. G. Betty, D.D.S., Cincinnati, Ohio."

This little work on craniology is the result of much thought, and study, and investigation by our friend, Dr. Betty. He has been for years greatly interested in this subject. In addition to much other study and investigation of this subject, he, a few months ago, spent nearly two months in examination of the large number of skulls in the Army Medical Museum. He made critical examinition of these and took accurate measurements with a view of, so far as possible, determining certain questions in regard to them and in regard to the teeth as well. Making notes, as will be seen in his tables, of the age and sex so far as possible. The measurements of the lower and upper jaws, noting as to presence or absence of the teeth; their irregularities as to whether there had been diseases of the teeth, either abscess or caries, and indeed all the facts pertaining to them that would be of interest and such as would illustrate the conditions of the teeth. In this he has done good work for the profession.

The best way for each and every one to become familiar with what the doctor has thus done is to procure the work and study closely.

This can be done by applying to him for the little volume.

